# Automatic Machine-Learning-Based Outcome Prediction in Patients With Primary Intracerebral Hemorrhage

**DOI:** 10.3389/fneur.2019.00910

**Published:** 2019-08-21

**Authors:** Hsueh-Lin Wang, Wei-Yen Hsu, Ming-Hsueh Lee, Hsu-Huei Weng, Sheng-Wei Chang, Jen-Tsung Yang, Yuan-Hsiung Tsai

**Affiliations:** ^1^Department of Diagnostic Radiology, Chang Gung Memorial Hospital, Chiayi, Taiwan; ^2^Department of Information Management, National Chung Cheng University, Chiayi, Taiwan; ^3^Department of Neurosurgery, Chang Gung Memorial Hospital, Chiayi, Taiwan; ^4^Chang Gung University College of Medicine, Taoyuan, Taiwan

**Keywords:** intracerebral hemorrhage, machine-learning, outcome prediction, random forest, auto-WEKA

## Abstract

**Background:** A predictive model can provide physicians, relatives, and patients the accurate information regarding the severity of disease and its predicted outcome. In this study, we used an automated machine-learning-based approach to construct a prognostic model to predict the functional outcome in patients with primary intracerebral hemorrhage (ICH).

**Methods:** We retrospectively collected data on demographic characteristics, laboratory studies and imaging findings of 333 patients with primary ICH. The functional outcomes at the 1st and 6th months after ICH were defined by the modified Rankin scale. All of the attributes were used for preprocessing and for automatic model selection with Automatic Waikato Environment for Knowledge Analysis. Confusion matrix and areas under the receiver operating characteristic curves (AUC) were used to test the predictive performance.

**Results:** Among the models tested, the random forest provided the best predictive performance for functional outcome. The overall accuracy for predicting the 1st month outcome was 83.1%, with 77.4% sensitivity and 86.9% specificity, and the AUC was 0.899. The overall accuracy for predicting the 6th month outcome was 83.9%, with 72.5% sensitivity and 90.6% specificity, and the AUC was 0.917.

**Conclusions:** Using an automatic machine learning technique to predict functional outcome after ICH is feasible, and the random forest model provides the best predictive performance across all tested models. This prediction model may provide information regarding functional outcome for clinicians that will help provide appropriate medical care for patients and information for their caregivers.

## Introduction

Recent advances in medical and interventional treatments have improved the prognosis of patients with ischemic stroke. However, despite the efforts related to the management of primary intracerebral hemorrhage (ICH) in the past decades, the beneficial effects of medical treatment and surgical intervention on the mortality and functional outcome of ICH patients have not been able to be demonstrated in recent trials (1, 2). Therefore, an outcome prediction model based on initial clinical presentations, laboratory data and imaging findings can ensure the optimal possible care by providing the physicians, relatives, and patients with information regarding to the severity of disease, potential risk of complications and predicted outcome (3). The ICH score is one of the most commonly used scales to grade severity of the disease (4). However, the ICH score, as well as other existing prognostication models of mortality and functional outcome have not been proven to be useful and beneficial. In addition to the diversification of the disease and its progression, the most important limitations of these predictive models are related to care limitations, such as the withdrawal of medical care, a do-not-resuscitate order (DNR), and comfort or terminal care (5). A prospective study showed that patients without early DNR orders had substantially lower 30-day mortality than predicted by the score (6). Another prospective study also questioned the validity of formal prognostic scales, as early subjective clinical judgment of physicians had a higher correlation with the 3-month outcome than that of the ICH score (7).

Machine learning is a type of artificial intelligence that learns patterns and rules from the given information. Machine learning has several advantages in detecting the possible interactions among many attributes and hence may be useful in clinical prediction and in the identification of novel prognostic markers (8). Recently, studies have applied machine learning to the severity or outcome prediction model for neurological disorders such as ischemic stroke (9, 10), aneurysmal subarachnoid hemorrhage (8), and traumatic brain injury (11). However, the application of machine learning in prediction of outcomes after ICH is still rare.

Waikato Environment for Knowledge Analysis (WEKA) machine learning software puts state-of-the-art machine learning techniques for a user friendly application (12). However, problems often encountered for novice users may include how to choose the best one from the dozens of machine learning techniques implemented in WEKA and to optimize each procedure's hyperparameter settings to achieve best performance (13). Auto-WEKA is developed to addresses these problems by treating the entire WEKA as one single, highly parametric machine learning framework and by using Bayesian optimization to find a strong instantiation for a given data set (13). This study aimed to apply machine learning using Auto-WEKA to predict the functional outcome in patients following ICH.

## Methods

### Patient Data Acquisition

The study was approved by the Chang Gung Memorial Hospital Institutional Review Board. The study included consecutive patients who were admitted due to primary ICH diagnosed by computerized tomography (CT) and enrolled in an integrated stroke study during January 2009 to December 2016. Patients with a history of head injuries, cerebral aneurysms, brain tumors, arteriovenous malformations, and subarachnoid hemorrhage were excluded. Patients who survived but dropped-out of the study before the 1st months after ICH were also excluded. Patients' clinical data were collected from the Health Information System. The data collected included the demographic data and the results of initial assessment (including vital signs, imaging findings, and laboratory tests). The demographic attributes included age, medical history of hypertension and diabetes (DM), blood pressure, and level of consciousness. The radiographic attributes included volume and location of hematoma, presence of intraventricular hemorrhage, ventricle compression, and midline structure shift. The volume of the hematoma was manually measured on the CT images using ImageJ software (https://imagej.nih.gov/ij/) by a trained research assistant. Laboratory attributes included the serum glucose level, aspartate aminotransferase (AST), alanine aminotransferase (ALT), blood urea nitrogen (BUN), creatinine (Cr), the BUN/creatinine ratio, glycosylated hemoglobin (HbAlc), the complete blood count (CBC), triglyceride (TG), total cholesterol, C-reactive protein (CRP), uric acid (UA), prothrombin time (PT), activated partial thrombin time (APTT), and hypersensitive CRP (hs CRP), determined at the first evaluation.

### Outcome Assessment

The primary outcomes of interest were the functional outcome measured with the modified Rankin scale (mRS) at the 1st and 6th months. Patients expired at the time outcome was measured (either at the 1st or 6th months) was scored as mRS of 6. Patients with mRS of 0, 1, and 2 were defined as having a good outcome, while those with mRS above 2 were defined as having a poor functional outcome.

### Construction of Predictive Models

**Figure 1:**
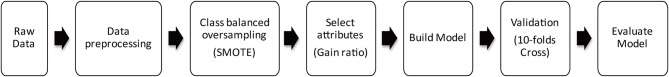
Machine learning method to predict the functional outcome in ICH patients.

All experiments were performed using Auto-WEKA software (https://www.cs.ubc.ca/labs/beta/Projects/autoweka/#). Figure 1 shows the steps of the modeling process used in this study.

#### Class-Balanced Oversampling

Machine learning algorithms have trouble learning when some classes dominate others; hence, oversampling and undersampling techniques are used to adjust the class distribution of the data set. In this study, we used the synthetic minority oversampling technique (SMOTE), in which the minority class is oversampled by creating synthetic examples rather than by oversampling with replacements (14).

#### Attribute Selection

Selection of attributes used for training is important for building a good model. This process applies a certain degree of cardinality reduction to reduce the number of attributes used because selecting the most important attributes can improve the accuracy of the model (15). Another advantage of the attribute selection is the reducing of processing time and space needed to build the model. In this study, the InfoGain module of Auto-WEKA was used for attribute selection. The information gain for each attribute was calculated using the ranker search method. The information gain measure is biased toward tests with many outcomes and prefers to select attributes with a large number of valid values (16). To reduce the effect of the bias due to the use of information gain, a technique known as the gain ratio was developed by the Australian academic Ross Quinlan (17). The gain ratio modulates the information gain to allow for the breadth and uniformity of attribute values for each attribute (18).

#### Building and Selection of the Machine Learning Models

In this study, several machine learning methods were evaluated and compared with Auto-WEKA to select the model that achieves the best performance for outcome prediction. Using recent innovations in Bayesian optimization, Auto-WEKA provides a fully automated approach to more effectively identify the machine learning algorithms and the hyperparameter settings appropriate for their applications, hence improving the performance of algorithm. There are 39 machine learning methods supported currently by Auto-WEKA 2.0, and Auto-WEKA uses a sequential model-based algorithm configuration (SMAC) to determine the class with the best performance on the given data (13).

#### Model Evaluation and Validation

In this study, we used a 10-fold cross validation and confusion matrix to build and evaluate the accuracy of the modules. The original samples were partitioned into 10 subsamples of approximately equal size. One of the 10 subsample was used as the validation data set for testing the models, and the remaining nine subsamples were used as training data set. The cross-validation process was then repeated 10 times with one of the 10 subsamples used sequentially for each validation. The 10 results from each of the repeated validation were then averaged to produce a final estimation.

In the confusion matrix, true negative (*TN)* is the number of negative examples correctly classified as negative, true positive (*TP)* is the number of positive examples correctly classified as positive, false negative (*FN)* is the number of positive examples incorrectly classified as negative and false positive (*FP)* is the number of negative examples incorrectly classified as positive. The accuracy is the performance measure generally associated with machine learning algorithms and is defined as (14)

$$\begin{array}{lll} \text{Sensitivity} & = & \text{TP}/\left(\text{TP}+\text{FN}\right)\\ \text{Specificity} & = & \text{TN}/\left(\text{FP}+\text{TN}\right)\\ \text{Accuracy} & = & \left(TP+TN\right)/\left(TP+FP+TN+FN\right) \end{array}$$

Receiver operating characteristic curves (ROC) are based on the false positive (1-specificity) of the x-axis and the true positive (sensitivity) of the y-axis. The sensitivity is the probability that the result is correctly judged to be positive. The specificity is the probability that the result is correctly judged to be negative. The closer the curve is to the top and the left, the higher the sensitivity and the lower the false positive rate of the classifier; that is, the discriminating power of the tool is better. Generally, when judging the quality of the inspection tool, in addition to looking at the graph of the curve, the area under the curve (AUC) can also be used to determine the discriminating power of the ROC curve. The AUC value ranges from 0 to 1, and the higher the value is, the better the predictive accuracy.

## Results

A total of 333 patients with ICH were enrolled in this study. The baseline demographic data for all patients are presented in Table 1. Among the 333 patients enrolled, the functional outcome data were available for 307 patients after the 1st month and for 243 patients after the 6th month.

**Table 1:** Baseline characteristics.

	**1-month outcome**		**6-month outcome**	
	**Poor (*N* = 177)**	**Good (*N* = 130)**	**Poor (*N* = 112)**	**Good (*N* = 131)**
Age (year)	65.80 (14.36)	61.40 (13.47)	69.98 (12.89)	60.15 (13.87)
Gender (male)	86 (32.9%)	101 (28%)	57 (23.5%)	87 (35.8%)
HTN	130 (42.3%)	98 (31.9%)	79 (32.5%)	107 (42.8%)
DM	37 (12.1%)	27 (8.8%)	25 (10.3%)	18 (7.4%)
Respiration (min)	19.36 (2.81)	19.18 (1.76)	19.64 (3.22)	19.21 (1.77)
DBP (mmHg)	104.17 (21.89)	107.19 (19.06)	103.05 (21.51)	109.38 (18.99)
GCS	10.27 (4.09)	13.56 (2.79)	9.41 (4.23)	13.45 (2.81)
**Laboratory studies**				
ALT (U/L)	30.84 (26.51)	33.22 (23.36)	30.18 (27.16)	34.80 (24.65)
BUN/Cr >15	90 (34.6%)	50 (19.2%)	57 (28.1%)	57 (28.1%)
Chol (mg/dL)	170.58 (44.33)	178.57 (28.66)	166.34 (44.32)	178.30 (36.50)
WBC (1000/uL)	9.76 (5.83)	8.73 (3.22)	9.79 (4.62)	8.50 (3.16)
Hgb (g/dL)	13.58 (1.8)	14.21 (1.66)	13.40 (1.76)	14.18 (1.62)
Hct (%)	39.84 (4.63)	41.68 (4.5)	39.16 (4.50)	41.43 (4.34)
APTT (sec)	27.37 (6.54)	27.7 (3.13)	27.76 (7.90)	27.07 (2.64)
hsCRP (mg/L)	24.55 (40.98)	12.24 (37.71)	26.96 (49.13)	13.35 (21.02)
**Image finding**				
**Location of the hematoma**				
Left lobar	11 (3.6%)	7 (2.3%)	11 (4.5%)	5 (2.1%)
Right lobar	11 (3.6%)	10 (3.3%)	9 (3.7%)	9 (3.7%)
Left thalamus	26 (8.5%)	14 (4.6%)	18 (7.4%)	13 (5.3%)
Right thalamus	29 (9.4%)	24 (7.8%)	15 (6.2%)	25 (10.3%)
Cerebellar	7 (2.3%)	10 (3.3%)	5 (2.1%)	7 (2.9%)
Brain stem	3 (1.0%)	1 (0.3%)	2 (0.8%)	2 (0.8%)
Left Basal ganglia	52 (16.9%)	36 (11.7%)	29 (11.9%)	38 (15.6%)
Right Basal ganglia	54 (17.6%)	27 (8.8%)	34 (14%)	34 (14%)
Other	0 (0.0%)	1 (0.3%)	0 (0.0%)	1 (0.4%)
IVH	83 (27.0%)	33 (10.7%)	58 (23.9%)	32 (13.2%)
Midline shift	106 (34.5%)	30 (9.8%)	65 (26.7%)	43 (17.7%)
Ventricle compression	109 (35.5%)	37 (12.1%)	66 (27.2%)	46 (18.9%)
ICH volume (cm^3^)	25.59 (31.54)	10.92 (13.94)	25.15 (24.53)	12.26 (13.99)

### Ranking of Attribute Importance

**Figure 2:**
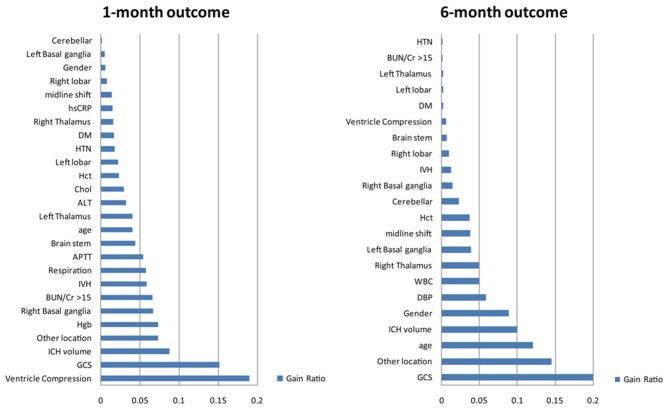
Selected attributes for building the models for predicting outcome after ICH. The information gain method was used to identify the most important attributes that significantly contribute to the accuracy of the models. Furthermore, the process of selection of attributes can help identify and remove irrelevant attributes by ranking all attributes based on their importance. The top 22 attributes were selected and included in the final model. These attributes are listed: GCS, Glasgow Coma Scale; HTN, hypertension; BUN/Cr >15, the ratio of blood urea nitrogen to creatinine exceeds 15; DM, diabetes mellitus; APTT, activated partial thromboplastin time; DBP, diastolic blood pressure; Hgb, hemoglobin; WBC, white blood cell; IVH, intraventricular hemorrhage; Hct, hematocrit; ALT, alanine aminotransferase; Cr, creatinine; hsCRP, high-sensitivity C-reactive protein; TG, triglyceride; R, right; L, left.

This study used the InfoGain module of Auto-WEKA to select and to rank the importance of the attributes for the functional outcome after the 1st and 6th months. The results are shown in Figure 2. There were 26 attributes selected based on their gain for the 1st month, including patient demographics (age and gender), GCS and respiration rate on admission, cardiovascular risk factors (hypertension and diabetes), labs on admission (serum CRP, ALT, Hgb, the BUN/Cr ratio, cholesterol, Hct, and APTT) and CT findings (locations of the hematoma, volume of the hematoma, ventricle compression, IVH, and midline shift). There were 22 attributes selected based on their gain for the 6th month which were mostly overlapping with that of the 1st month except for the white count level.

### Selection of the Best Classifier

**Figure 3:**
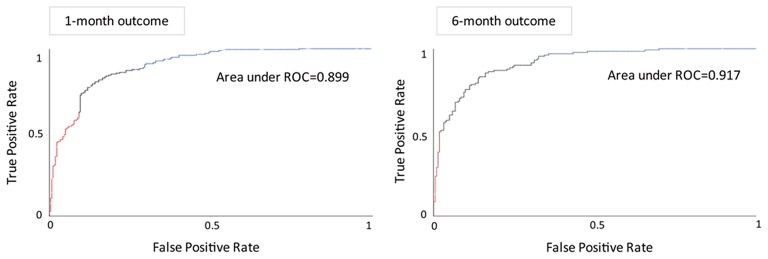
Receiver operating characteristic curves and areas under the curves of the predictive models for the functional outcome after the 1st month **(left)** and 6th month **(right)**.

We used Auto-WEKA to select the best predictive algorithm. The models were evaluated using 10-fold cross validation according to the AUC metric. The random forest was selected as the best classifier for predicting the outcomes at both the 1st and 6th months after ICH. The accuracy for predicting the 1st month outcome was 83.1%, with 77.4% sensitivity and 86.9% specificity, and the AUC was 0.899. The accuracy for predicting the 6th month outcome was 83.9%, with 72.5% sensitivity and 90.6% specificity, and the AUC was 0.917 (Table 2; Figure 3).

**Table 2:** Using Auto-WEKA to select the best predictive algorithm.

**Time after ICH**	**Case number**	**Best algorithms**	**Sensitivity**	**Specificity**	**Accuracy**	**AUC**
1-month	307	Random forest	0.774	0.869	0.831	0.899
6-month	243	Random forest	0.725	0.906	0.839	0.917

## Discussion

In this study, we developed an automatic machine learning model for predicting the outcome of ICH patients. We analyzed the medical history, laboratory and imaging findings of 333 ICH patients by reviewing their medical records. The results showed that random forest was the most accurate algorithm to predict the functional outcome using attributes that are automatically selected with information gain. The information about the severity of ICH and its predicted outcome is crucial for decision making and for possible long-term care. Furthermore, to find-out the associated risk factors which have not been noticed in the conventional scoring system might provide a clue for future study regarding to the patient management. Using Auto-WEKA software, we can simultaneously develop and evaluate the performance of several different machine learning techniques. This approach is effective to overcome one of the most challenging part of the machine learning process that requires iterative and explorative experiments to build or to select a model that can achieve the best accuracy and is more suitable for general users.

In practice, the available past medical history and admission data about the disease severity of ICH patients can provide important information for predicting outcomes. The most widely used risk stratification scale for ICH is the ICH score. Independent predictors of 30-day mortality in the ICH score are low score on GCS, greater ICH volume, older age, infratentorial location of the hematoma, and intraventricular extension of the hematoma (4). Notably, these 5 variables are all included in our model for predicting 1-month outcome using the information gain method. Although our model was designed to predict the functional outcome instead of mortality, our results confirm the importance of these factors. Other factors, such as evidence of subfalcine brain herniation (midline structure shift) on CT, history of DM and HTN, detailed locations of the hematoma and laboratory studies, such as Hgb, hsCRP, cholesterol, WBC, and the BUN/Cr ratio, are also associated with outcome. Using machine learning techniques, we may be able to identify the factors that have been previously neglected and to develop new treatment methods to improve outcomes according to these factors. Furthermore, the developed model in this study is estimated from a small number of attributes which can be recorded easily without extra clinical loading, yet it can provide an optimal prediction of functional outcome. Thus, the model is simple, reliable, and can be easily adopted in clinical practice.

Among the 39 machine learning methods tested by Auto-WEKA, the random forest prediction model was the most accurate for predicting outcome after both the 1st and 6th months. Random forest is a machine learning technique that based on forming multiple decision trees by a random selection of samples. The decision tree learns decision rules extracted from the features of data. The deeper the tree is, the more complex the decision rules are, resulting in a better fitting of the model. For the classification of a new record from an input vector, the input vector is put on every tree in the forest. Each tree votes for a specific class label and the one that gets most votes over all the trees in the forest will be the final class label (19, 20). Random forests overcome the problem of overfitting decision trees. However, as we know, the performance of a machine learning algorithm can vary from one data set to another and there are no algorithms that can achieve good performance of all possible learning problems. In fact, practitioner without enough experience of machine learning techniques might choose a complicated and inappropriate machine learning algorithm that could lead to poor results, even with great effort and loss of time. To solve this problem, several automatic machine learning techniques, including Auto-WEKA, have emerged as a new subarea in machine learning (13, 21). These tools can enable easier and faster deployment of machine learning tools across institutions, efficiently validate and test the performance of deployed solutions, and make researchers focus more on problems with more applications and clinical value.

Our study nonetheless had several limitations. First, some patients with very large hematomas or very critical conditions were not included in the study because of the family's decision not to be followed-up or participate in the study, which may restrict the generalizability of the results. Early withdrawal of care and self-fulfilling prophecies may also affect the accuracy of this predictive model. Second, although the performances of the algorithms are good, the sample size is relatively small. Studies with larger samples may result in a higher predictive power. Third, we did not enroll the information of early hematoma growth and extension of edema which are both important for outcome into this model. Finally, external validation to test the generalizability and to exclude the institutional bias is lacking.

In conclusion, the prediction of functional outcome after ICH is a challenging undertaking. Our study using an automatic machine-learning-based approach showed promising results. The random forest algorithm provides the best predictive performance across all tested models for both the 1st and 6th month functional outcomes with considerable accuracy. This prediction model may provide information regarding functional outcome for clinicians to provide appropriate medical care for patients and information for their caregivers.

## Data Availability

The datasets generated for this study are available on request to the corresponding author.

## Author Contributions

H-LW and W-YH: literature review, data processing, manuscript writing, and tables making. M-HL and J-TY: clinical data collection and cases review. S-WC and H-HW: imaging analysis. Y-HT: manuscript review and editing.

### Conflict of Interest Statement

The authors declare that the research was conducted in the absence of any commercial or financial relationships that could be construed as a potential conflict of interest.
